# Anti-Proliferative Activity of Triterpenes Isolated from *Clinicanthus nutans* on Hep-G2 Liver Cancer Cells

**DOI:** 10.31557/APJCP.2019.20.2.563

**Published:** 2019

**Authors:** Khairun Najwa Zakaria, Azura Amid, Zubaidah Zakaria, Parveen Jamal, Azli Ismail

**Affiliations:** 1 *Department of Biotechnology Engineering, Faculty of Engineering,*; 2 *International Institute for Halal Research and Training, Level 3, KICT Building, International Islamic University Malaysia,*; 3 *Haematology Unit, Cancer Research Centre, Institute for Medical Research, Kuala Lumpur, Malaysia. *

**Keywords:** Clinicanthus nutans, anti-proliferative, triterpenes, liver cancer

## Abstract

**Problem statement:**

*Clinicanthus nutans *has been used by Malaysian since long time ago. It is used to treat many diseases including cancer. Many studies carried out on its crude extract but no clear report on the specific secondary metabolites responsible for its nature in treating selected diseases.

**Objective:**

This study aims to confirm the practice carried out by many people on the usage of *Clinicanthus nutans *in treating cancer.

**Methods:**

*C. nutans* leaves were extracted by methanol. Thin layer chromatography was used to identify the suitable solvent for fractions separation. The fractions were then separated at larger volume using gravity column chromatography. Each fraction was tested on its anti-proliferative activity on Hep-G2 liver cancer cells by MTT assay. The phytochemical screening was carried out to identify the bioactive compound based on qualitative analysis.

**Results:**

The fraction 2 (F2) of *C. nutans* showed the lowest IC_50_ value of 1.73 µg/ml against Hep-G2 cancer cells, and it is identified as triterpenes.

**Conclusion:**

The fraction F2 identified as triterpenes isolated from *C. nutans* has potential as an anti-proliferative agent against liver cancer.

## Introduction

*Clinacanthus nutans (C. nutans)* Lindau, or commonly known as Sabah Snake Grass or Belalai Gajah in Malay is one of the plants that is known for its high medicinal values. *C. nutans* belongs to the family of Acanthaceae. It is a small shrub, native in tropical weather and can be found easily in Malaysia, Thailand, and Indonesia (Alam et al., 2016). It is normally consumed by Malaysian especially the Chinese ethnic because they believed it might prevent them from cancer. Interestingly, few scientific studies were carried out to confirm these.

Arullapan et al., (2014) conducted a study to evaluate the *in vitro* cytotoxic, antioxidant and antimicrobial activities of *C. nutans* leave extracts and its semi-fractions. In that study, HeLa (cervical cancer) and K-562 (leukemia) cell lines were used. The crude extract of *C. nutans* leaves (0.2 to 10 µg/mL) was tested against HeLa and K-562 cell lines using 3-(4,5-dimethylthiazol-2-yl)-2,5-diphenyltetrazolium bromide (MTT) assay, and antioxidant activity using 1,1-diphenyl-2-picrylhydrazyl (DPPH) assay. The results showed that the fractions isolated from ethyl acetate leaf extract demonstrated cytotoxic, antioxidant and antimicrobial activities. The IC_50_ values against HeLa and K-562 cell lines during cytotoxicity assay were 18.0 and 20.0 µg/mL, respectively. However, there is no study reported liver cancer treated with *C. nutans* extract.

The Hep-G2 cell line was originally established in 1979 by Barbara Knowles and colleagues (López-Terrada et al., 2009). Hep-G2 liver cancer cell line was derived from 15 years old Caucasian American white male, whose well-differentiated Hepatocellular carcinoma, the most common type of liver cancer (ATCC, 2003). Hep-G2 is adherent, epithelial-like cells growing as monolayers and in small aggregates. These cells are epithelial in morphology, have a modal chromosome number of 55, and are not tumorigenic in nude mice (ATCC, 2003). Because of their high degree of morphological and functional differentiation* in vitro*, Hep-G2 cells are a suitable model to study human liver diseases (Mersch-Sundermann et al., 2004).

## Materials and Methods

*Raw Material*

The leaves of *C. nutans* were used for this project. The *C. nutans* leaves were harvested from the self-plant garden in Nilai, Negeri Sembilan, Malaysia. A plant sample deposited in the herbarium at the Kulliyyah of Architecture and Environmental Design (KAED), International Islamic University Malaysia with voucher number KAED/HBL/S1A047/2018/706. The name of the plant was checked on www.theplantlist.org 

*Cell Line*

This study used Hep-G2 liver cancer cell line (originally from ATCC No: HB-8065; obtained from Institute Medical Research, Kuala Lumpur) as the experimental cancer cells. It was kept in liquid nitrogen until further use. 

*Chemicals and Reagents*

All the chemicals and reagents used in this project are laboratory grade. They were purchased from Merck (USA), Sigma-Aldrich (USA), GIBCO (USA) and Fisher Scientific (USA). 

*C. nutans Extraction and Fractionation*

The freshly harvested of *C. nutans* leaves were air dried for 72 h. The dried leaves were grounded into a fine powder to increase the surface area. The *C. nutans* extract (25 g) was then prepared using the maceration process with methanol (250 mL) for 72 h at room temperature under constant shaking. The extract collected was wrapped with aluminum foil, to keep them in the less-light exposure condition and stored at 4 ͦC chiller until use (P’ng et al., 2013). The *C. nutans* extract was separated by thin layer chromatography (TLC) and next by column chromatography. TLC plates (20 x 10 cm) coated with silica gel (Merck, Germany) and ultraviolet (UV) lamp for spot detection was used in this experiment. Samples (20 µL) were spotted on TLC plates using a syringe, two centimeter above the bottom edge and allow to develop using mobile phase until it reached the solvent front. The plates were then removed, dried, observed under the UV light and finally, the RF-values were calculated. Best separations were achieved with hexane: ethyl acetate (7:3). Column chromatography was performed using the identified mobile phase to get more volume of each fraction. 

*Anti-proliferative Test*

The Hep-G2 cells were cultured in DMEM supplemented with 1 % antibiotics (100 U/mL penicillin, 100 µg/mL streptomycin) and 10% Fetal Bovine Serum (FBS) and incubated at 37^o^C in a 95% air and 5% CO_2_ atmosphere. Once confluence, Hep-G2 cells were trypsinised, re-suspended in media and counted using a haemocytometer. The cells were then diluted to 1.0 x 10^5 ^cells/mL and plated in 100 mL of medium/well in 96-well plate. The cells were then incubated for 24 h. After 24 h, media was removed and the cells were then exposed to the four *C. nutans* leaves extract active fractions in the presence of various concentrations at eight concentration intervals (between 0.78 and 100 µg/mL) for another 24 h at 37 ͦC. The sample solutions were then removed and washed with phosphate buffer solution (pH 7.4). A volume of 20 µL/well of MTT at 5 mg/mL concentration was added to each well. The assay then was incubated for 4 h at 37^o^C. The amount of formazan was determined by measuring the absorbance at 570 nm. The higher the absorbance value, the more converted formazan is present, and so the higher the number of viable cells. This method was carried out in triplicates. The data obtained using microplate reader (ThermoFisher) was used to calculate the percentage of viable and unviable cells. The formulas used (Wan Chik et al., 2010) were in equations (1) and (2) below. 

Equation 1 $$\mathrm{\% cell growth}=\frac{\mathrm{OD sample}}{\mathrm{OD control}}\times 100$$

% cell killed=100-% cell growth

 Equation 2

Where the value of the optical density (OD) sample is the OD for the cells treated with active fractions and the OD control is the OD for cells without treatment.

*Phytochemical Screening*

Phenol was detected using Ferric Chloride test (Ahluwalia and Raghav, 1997). Flavonoids were detected using Alkaline Reagent test (Evans, 2002), and Ammonia test (Harborne, 1998). Alkaloids were detected by Wagner’s test (Harborne, 1998). Saponins were detected by Sulphuric Acid Reaction test and Emulsion Formation test (Brain and Turner, 1975). Terpenoids were detected by Salkowski test (Ayoola et al., 2008) and Liebermann-Burchard test (Kumar et al., 2010). Fixed oils and fats were detected by oil stain test (Kokate et al., 2008) and finally, Cardiac Glycosides was detected by Keller Killiani test (Ayoola et al., 2008).

## Results

*C. nutans* Extraction and Fractionation 

Table 1 presents the results for extraction and fractionation. The hexane/ethyl acetate at a ratio of 7:3 resulted in four separate spots on the TLC plate. For further separation by open column chromatography, ratio 7:3 has been chosen as mobile phase due to the suitable RF-value showing well-separated spots with RF-value from 0.34 to 0.93, polarity index of 1.86 suggested that there is a preeminent separation of the *C. nutans* extract. This is because it would be easier to collect the fractions during the open column chromatography analysis when the spots are not close to each other. 

For column chromatography, separated fractions were collected in 15 mL centrifuge tubes and were labeled with number 1 to 77 and proceed to vacuum concentrator. Again, TLC was used to monitor the effectiveness of the separation. Fractions with similar RF-value were group together. In this case, based on the RF-value, tube number 1 to 6 were group together and named as compound/fraction 1 (F1) (RF-value = 0.93), tube number 19-29 were fraction 2 (F2) (RF-value = 0.83), tube number 40-46 were fraction 3 (F3) (RF-value =0.59) and tube number 70-77 were fraction 4 (F4) (RF-value = 0.34). At this point, the four spots showed different RF-values because they might represent different compound, which at the same time having different polarity. Thus, phytochemical screening process takes part for further validation.

*Phytochemical screening of C. nutans fractions*

The compounds identified via phytochemical screening are tabulated in Table 2. They were evaluated based on colour formation, precipitate, froth formation, visibility of the ring formation between two layers and stain formation. The fractions revealed the presence of carotenoids (F1), triterpenes (F2), saponins (F4) and cardiac glycosides (F3) (Table 2) as secondary metabolites that might be responsible for the anti-proliferative activity of the cells. Phenols, flavonoids, alkaloids, fixed oils, and fats were not detected in the fractions.

*IC*_50_ *of C. nutans Fractions by MTT Assay*

This assay was carried out to identify the active fraction which has the anti-cancer activity and determine its IC_50_. In this experiment, Hep-G2 cells were treated with all four active fractions (F1 – F4) and taxol used as a standard at eight concentration intervals, ranging from 0.781 to 100 µg/mL and 0.0156 to 2 µg/mL, respectively. The active fraction that is capable of inhibiting the cells growth can be assumed to be useful in the treatment of liver cancer.

**Figure 1:**
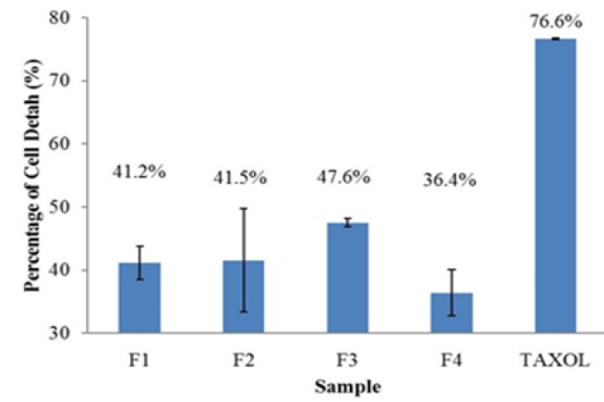
Percentage of Hep-G2 Cells Death after Treated with Isolated Fractions (F1-F4) from *C. nutans *Leaves Extract (1.56 µg/mL), and Taxol (2 µg/ml) Presented with the Standard Deviation Obtained from Three Independent Experiments

For screening purpose, the percentage of cell death of all active fractions were plotted in a bar graph at the same concentration (1.56 µg/mL), and taxol at 2 µg/mL. Referring to Figure 1, among all those four fractions at concentration 1.56 µg/mL, active fraction 3 (F3) which is also preliminarily identified as cardiac-glycosides (Table 2) demonstrated the highest percentage of Hep-G2 cell death with 47.6 %.

The data for the MTT assay were plotted in the graph of percentage of viable cells versus concentration (µg/mL) to identify the IC_50_ of each fraction and taxol. The result (Table 3) showed that F2 gave the lowest IC_50_ value (1.73 µg/ml), while F3 gave the IC_50_ of 4.51 µg/ml. The results suggesting that F2 is more potent than F3. When treated with adherent Hep-G2 cells, taxol gave the IC_50_ value of 0.11 µg/mL. Thus, active fraction F2 is considered to demonstrate a good result in this experiment.

## Discussion

RF-value is equal to the distance traveled by the substance divided by the distance traveled by the solvent. Typically, an effective solvent is one that gives RF- value in the range of 0.5-1.0 (Touchstone, 1992). It is also reported that the solvent system with the highest polarity will make the components in the mixture move along with the solvent and caused no separation observed and the RF-value will be too large. Thus, from the results in Table 1, the solvent system of hexane : ethyl acetate with ratio 7 : 3, polarity index value of 1.83 which gave the best readings of RF-values, ranging from 0.34 to 0.93 had been selected for further use in column chromatography. Later, column chromatography (CC) was used to collect the active fractions from crude of *C. nutans* leaves extract by gravity force. CC applied the same principles as TLC but performed at larger volume (Chik et al., 2011; Tolar and Neglia, 2003). Subsequently, phytochemical screening of the collected four active fractions was carried out to preliminary identify the type of secondary metabolites separated and isolated by CC. According to Table 2, terpenoids were isolated and collected in this experiment. This finding was supported by (Wan Chik et al., 2010; Çitoğlu and Acıkara, 2012), because of ethanol, methanol, and water led to the extraction of highly oxygenated namely polar triterpenes as well as triterpenoid and sterol glycosides. Besides, Tsado et al., (2016) used the same method and successfully extracted terpenoids from *Newbouldia laevis and Crateva adansonii* leaves using methanol as a solvent. Secondly, based on the phytochemical screening result (Table 2) shows that the presence of carotenoid in F1, triterpenes in F2, saponin in F3 and cardiac glycosides in F4. Referring to (Yamunadevi et al., 2011; Harborne, 1998), the combination of hexane: ethyl acetate is commonly used as the mobile phase to isolate and purify terpenoids group. Yamunadevi and co-workers (Yamunadevi et al., 2011) reported that 27 different types of terpenoids were detected by HPLTC, with RF- value range of 0.31 to 0.97. This information supports our current findings, which high possibility that the F2 is in terpenoids group.

**Table 1:** Rf value with Different Solvents Ratio for Hexane/ethyl Acetate

M2 solvent ratio	No. of spots detected	Polarity index of mixture (PI mix)	Retardation factor (Rf) value.
9:01	1	0.62	0.35
8:02	4	1.24	0.22
			0.39
			0.53
			0.79
7:03	4	1.86	0.34
			0.59
			0.83
			0.93
1:01	0	3.1	-
3:07	0	4.34	-
2:08	0	4.96	-
1:09	0	5.58	-

**Table 2:** Phytochemical Evaluation of the Active Secondary Metabolites Presents in the Isolated Active Fractions (F1 – F4)

Class of compound	Test	Specific constituents	Result			
			F1	F2	F3	F4
Phenolic compound	Ferric chloride test	Phenols	-	-	-	-
	Alkaline reagent test	Flavonoids	-	-	-	-
	Ammonia test	Flavonoids	-	-	-	-
Alkaloids	Wagner’s test	Alkaloids	-	-	-	-
Terpenoids	Emulsion formation test	Saponin	-	-	+	++
	Liebermann-Burchard test	Triterpenes	-	+	-	-
	Salkowski test	Triterpenes	-	+	-	-
	Modifies Brontranger’s test	Cardiac-glycosides	-	-	+	-
	Legal’s test	Cardiac-glycosides	-	-	+	-
	Sulphuric acid test	Carotenoids	+	-	-	-
Carbohydrates		Carbohydrates	-	-	-	-
Fixed oils and fats	Stain test	Oil and fats	-	-	-	-

Positive (+) and negative (-) signs showed the presence and absence of metabolites in the fractions, with (++) strong colour and (+) mild colour.

**Table 3:** the IC_50_ Value of Each Fraction and Taxol

Sample	F1	F2	F3	F4	Taxol
IC_50_ (µg/mL)	4.68	1.73	4.51	10.5	0.11

The current study shows the interest of valuable natural substance, such as terpenoids has been increasing day by day (Yamunadevi et al., 2011). Terpenoids are defined as secondary metabolites with molecular structures containing carbon backbones made up of isoprene (2-methylbuta- 1, 3-diene) units (Harborne, 1998). More than 36 000 terpenoids compounds have been identified, making terpenoids the largest class of plant metabolites. In particular, terpenoids contained in many herbal plants, with several kinds of terpenoids are already available for pharmaceutical applications today, such as taxol and artemisinin as a chemotherapy drug and malaria medicines, respectively (Yamunadevi et al., 2011). Generally, plant terpenoids are well-known to possess useful aromatic qualities (Harborne, 1998). It has been used traditionally in herbal remedies and still under investigation of the pharmaceutical function, such as antibacterial, anticancer and other therapeutic properties. Until now, there is no such study which carried out specifically on terpenoids isolated from *C. nutans* leaves extracted as an anticancer agent. However, the crude extract of this plant shows their anticancer properties. Fong (2015) reported that the methanol and water extracts of *C. nutans* leaves were cytotoxic against D24 and MM418C1 melanoma cells, MCF-7 and BT474 breast carcinoma cells. Other pharmacology properties of crude *C. nutans* leaves extract such as antioxidant activity, anti-proliferative activity, anti-tumour, and cytotoxic agents. This suggests that the extracts may contain the anti-cancer compound. Based on these results, terpenoids may be considered as a promising anticancer agent present in the *C. nutans* leaves extracts. The cytotoxicity of the fractions were further defined by determining the half maximal inhibitory concentration (IC_50_). The four active fractions from *C. nutans* leave extract were used from this experiment onwards. 

Initial yellow in colour of MTT assay is reduced to purple formazan in the mitochondria of living cells. The reduction will take place only when the mitochondrial reductase enzyme is active. Therefore, the conversion can be directly related to the number of viable/living cells. Through this experiment, the effectiveness of the sample in causing death to the cells can be measured by the amount of purple formazan produced after the sample treated with active fractions or taxol compared to the amounts of untreated cells (Freshney, 2010). Among all the four active fractions of *C. nutans* leaves extract, F3 showed the highest percentage of Hep-G2 cells death (47.6 %) at 1.56 µg/mL, while taxol gave 76.6 % at concentration 2 µg/mL. However, active fraction F2 exhibited the highest percentage of Hep-G2 cancer cells inhibition in almost every concentration tested (4 over 6 concentration tested; 1.56, 3.13, 6.25 and 25 µg/ml) than other active fractions.

Later, the MTT assay results on adherent Hep-G2 cells (Figure 1) shows that F2 gave a reasonable and exceptional effect against Hep-G2 liver cancer cells because the value of F2 IC_50_ is in the acceptable range of suitable as cytotoxicity agent. According to the US National Cancer Institute (NCI) plant screening program, plant extract is generally considered to have *in vitro* cytotoxicity activity if the IC_50_ value is less than 20 µg/mL (Boik, 2001). The F2 fraction gave an IC_50_ value higher than taxol but still lesser than 20 µg/mL, supporting a milder effect of native-based product compared to highly, toxic taxol. Several papers on *C. nutans* leaves extract showed that the crude extract possesses potential anticancer agents towards cultured cancer cell lines such as HeLa and K-562 cells (Arullappan et al., 2014) and Raji lymphoma cells (Yong et al., 2013) but those studies did not identify the type of secondary metabolites responsible for the anti-cancer activity. While, from a phytochemical screening test of this study, an isolated active fraction from crude *C. nutans* leaves extract, F2 showed positive results towards Lieberman-Burchard reagent, indicated that this compound possibly triterpenoid. Triterpenoid is a well-known secondary metabolite compound that possesses such anticancer properties. Triterpenoid derived from avocado showed a positive result on Hep-G2 liver cancer cells, suggesting the *in vitro* cytotoxicity of triterpenoid (Abubakar et al., 2017). The same study also reported that triterpenoid was also safe to normal Vero cells (Abubakar et al., 2017). Many triterpenoids have various biological activities, e.g., anti-tumour, anti-viral, anti-bacterial, virostatic, anti-ulcerogenic, anti-inflammatory (Dzubak et al., 2006). Triterpenoids are used for medicinal purposes in many Asian countries for anti-inflammatory, analgesic, antipyretic, hepatoprotective, cardiotonic, sedative and tonic effects (Moses et al., 2014). An increasing number of triterpenoids have been reported to exhibit cytotoxicity against a variety of *in vitro* cancer cells (MCF-7 breast cancer cells, Hep-G2 liver cancer cells) without manifesting any toxicity in normal cells (Vero cells) ( Moses et al., 2014).

In conclusion, the triterpenoids isolated from *C. nutans* were able to show the positive anti-proliferative effect on Hep-G2 liver cancer cells. Thus, *C. nutans* is a potential alternative or complementary medicine for treating liver cancer in a human.
